# Characterization of the plastid genome of the monotypic genus *Bryocarpum* (Primulaceae)

**DOI:** 10.1080/23802359.2019.1666043

**Published:** 2019-09-20

**Authors:** Ting Li, Feng Song, Xun Yuan, Tongjian Liu, Haifei Yan

**Affiliations:** a Key Laboratory of Plant Resources Conservation and Sustainable Utilization, South China Botanical Garden, Chinese Academy of Sciences, Guangzhou, China;; b University of Chinese Academy of Sciences, Beijing, China;; c Key Laboratory of Biogeography and Bioresource in Arid Land, Xinjiang Institute of Ecology and Geography, Chinese Academy of Sciences, Urumqi, China;; d College of Life Sciences, South China Agricultural University, Guangzhou, China

**Keywords:** *Bryocarpum himalaicum*, plastome, Illumina sequencing, phylogeny

## Abstract

*Bryocarpum* Hook. f. & Thoms., a monotypic genus of Primulaceae, is narrowly distributed in the eastern Himalayas. In this study, the complete plastid genome of *Bryocarpum himalaicum* was characterized by Illumina paired-end sequencing reads. The plastid genome of *B. himalaicum* is 153,398 bp in length, including large single copy (LSC) region of 84,446 bp, small single copy (SSC) region of 17,564 bp, and two separated inverted regions (IRs) of 25,694 bp, each. It encodes 110 genes, of which 79 are protein-coding genes, 4 are rRNA genes, and 27 are tRNA genes. The phylogenetic result indicates *B. himalaicum* is sister to the genus *Primula*.

*Bryocarpum* Hook. f. & Thoms. is a monotypic genus of Primulaceae with unique characters, such as 7-merous flowers and circumscissile capsule (Hu and Kelso 1996). *Bryocarpum himalaicum* Hook. f. & Thoms., the only species of *Bryocarpum*, is narrowly distributed in eastern Himalayas (Bhutan, Nepal, Sikkim, and SE Xizang of China; Hu and Kelso 1996). Morphologically, the genus *Bryocarpum* resembles *Omphalogramma* Franch. (Hu and Kelso 1996). In this study, we sequenced and characterized the plastid genome of *B. himalaicum* for better understanding of its phylogenetic position.

Fresh leaves of *B. himalaicum* were collected from Motuo of Xizang, China (95°42′30″E, 29°45′22″N), and dried in silica-gel for DNA extraction with the modified CTAB method (Doyle and Doyle 1987). The voucher specimen (No. Xu, Liu and Huang 150215) was deposited at the herbarium of South China Botanical Garden. The yielded DNA was sheared into *ca*. 500 bp fragments for paired-end (2 × 150 bp) library construction. The library was sequenced by Illumina Hiseq X Ten platform at Beijing Genomics Institute (Shenzhen, China). About 3 Gb of paired-end clean data were obtained after removing low-quality reads and adaptor sequences. The complete chloroplast genome of *B. himalaicum* was assembled by NOVOPlasty 2.6.3 (Dierckxsens et al. 2017). Gene annotation was performed in Geneious ver. 11.0.2 (Kearse et al. 2012) and tRNA genes were predicted by using ARAGORN (Laslett and Canback 2004). Sequences of the 79 shared protein-coding genes were extracted from the plastomes of the 12 taxa within Primulaceae (Figure 1). Sequence alignment for each gene was performed using MAFFT (Katoh and Standley 2013) and concatenated into a single alignment. The maximum-likelihood tree from the concatenated alignment was constructed using RAxML (Stamatakis 2014) with GTRGAMMA model under the rapid bootstrap algorithm (1000 replicates).

**Figure 1.**
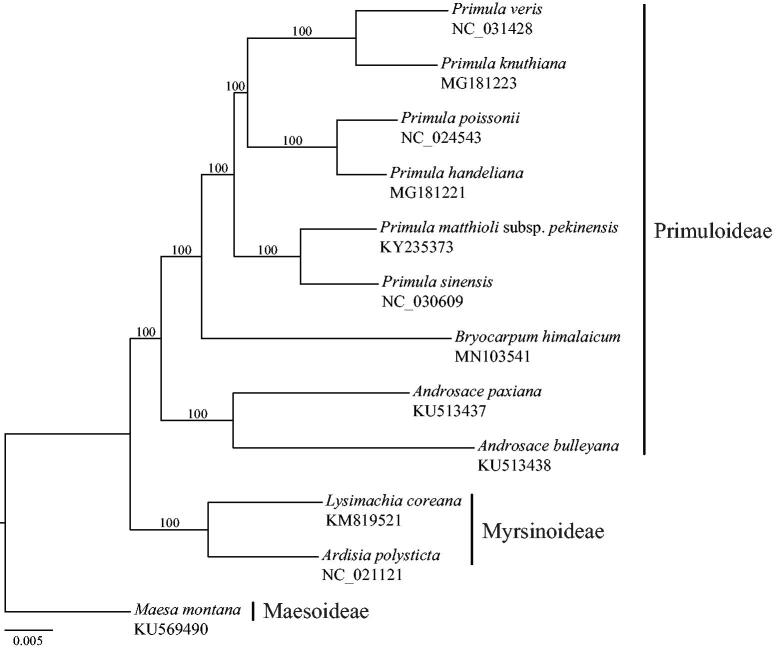
Maximum-likelihood tree based on 79 protein-coding genes from plastid genomes of Primuloideae. Taxa within Myrsinoideae and Maesoideae were selected as outgroups. Numbers on the branches are bootstrap support values based on 1000 replicates.

The *B. himalaicum* plastid genome was assembled as circular molecules and deposited in GenBank under accession number MN103541. The plastome is 153,398 bp long, including large single copy (LSC) region of 84,446 bp, small single copy (SSC) region of 17,564 bp, and two separated inverted regions (IRs) of 25,694 bp, each. The overall GC content of the genome is 36.9%, whereas the GC content in the LSC, SSC, and IR regions are 34.8, 29.9, and 42.7%, respectively. The plastome encodes 110 genes, of which 79 are protein-coding genes, 4 are rRNA genes, and 27 are tRNA genes. Eighteen genes (seven protein-coding, four rRNA, and seven tRNA) are duplicated in the IR regions. Sixty-one protein-coding genes and 21 tRNA genes are located in LSC region, while 12 protein-coding genes and 1 tRNA gene occur in the SSC region. Among 110 genes, 16 genes contain one intron and three genes (*clpP*, *rps12*, and *ycf3*) contain two introns. The monophyly of the subfamily Primuloideae is strongly supported (Figure 1). *Bryocarpum himalaicum* is sister to the genus *Primula*, which is consistent with the results of Martins et al. (2003) and Mast et al. (2006).
